# Systematic Analysis of a Pyroptosis-Related Signature to Predict the Prognosis and Immune Microenvironment of Lower-Grade Glioma

**DOI:** 10.3390/cells11243980

**Published:** 2022-12-09

**Authors:** Yongze He, Yuxiang Cai, Jinsheng Liu, Haixia Ding, Xiang Li, Sufang Tian, Zhiqiang Li

**Affiliations:** 1 Brain Glioma Center, Zhongnan Hospital of Wuhan University, Wuhan 430062, China; 2 Department of Pathology, Zhongnan Hospital of Wuhan University, Wuhan 430062, China; 3 Department of Oncology, Zhongnan Hospital of Wuhan University, Wuhan 430062, China

**Keywords:** pyroptosis, LGG, tumor microenvironment, LASSO, prognosis, immunohistochemical

## Abstract

Current treatments for lower-grade glioma (LGG) do not effectively improve life expectancy rates, and this is a major global health concern. Improving our knowledge of this disease will ultimately help to improve prevention, accurate prognosis, and treatment strategies. Pyroptosis is an inflammatory form of regulated cell death, which plays an important role in tumor progression and occurrence. There is still a lack of effective markers to evaluate the prognosis of LGG patients. We collected paraffin-embedded tissue samples and prognostic information from 85 patients with low-grade gliomas and fabricated them into a tissue microarray. Combining data from public databases, we explored the relationship between pyroptosis-related genes (PRGs) and the prognoses of patients with LGG and investigated their correlations with the tumor microenvironment (TME) by means of machine learning, single-cell, immunohistochemical, nomogram, GSEA, and Cox regression analyses. We developed a six-gene PRG-based prognostic model, and the results have identified CASP4 as an effective marker for LGG prognosis predictions. Furthermore, the effects on immune cell infiltration may also provide guidance for future immunotherapy strategies.

## 1. Introduction

Glioma is a frequently seen brain tumor type and is classified by the WHO into grades 1–4 [1]. Grade 2 and 3 tumors are considered lower-grade gliomas (LGGs), and grade 4 tumors are referred to as glioblastoma (GBM) [2]. LGGs have longer survival times than GBM (median survival times of 2–10 years vs. 14–16 months, respectively) [3]; however, LGGs are also prone to recurrence after surgery, and there is a risk of transformation to GBM [4]. The survival times of patients with LGG vary greatly among individuals with each histological subtype of glioma [5]. There is currently a shortage of effective markers for evaluating LGG patients’ prognostic outcomes [5,6], and this needs to be addressed, as identifying such markers could improve prognosis accuracy and patient quality of life.

Programmed cell death (PCD) represents the basic mode of destroying damaged or abnormal cells [7]. The known types of PCD include necrosis, apoptosis, necroptosis, autophagy, ferroptosis, and pyroptosis [8]. Pyroptosis differs from other PCDs and has the features of cell membrane pore generation, plasma membrane rupture, and cell swelling [9], causing proinflammatory factors (IL-1β and IL-18) and the cell contents to be produced in the extracellular space, thereby triggering an inflammatory response [10]. Among the major pathways, pyroptosis is induced by gasdermin D (GSDMD), which includes the inflammasome activation of caspase-1 (canonical pathway), the lipopolysaccharide (LPS) activation of caspase-4/5, and mouse caspase-11 (atypical pathway) [11]. Among the alternative pathways, the induction of pyroptosis by gasdermin E (GSDME) via CASP3 is the most important [12]. Additionally, the cleavage of gasdermin B (GSDMB) by granase B (GzmB) or granase A (GzmA) can also lead to pyroptosis [13].

Inflammatory factors and associated pathways involved in pyroptosis can be tightly associated with the occurrence and development of tumors and their resistance to chemotherapeutic drugs [14,15]. According to the latest articles, pyroptosis-related genes (PRGs) have important roles in various tumors, including lung adenocarcinoma (LUAD) [16], head and neck squamous cell carcinoma (HNSCC) [17], GBM [18], ovarian cancer (OC) [19], renal clear cell carcinoma (RCCC) [20], and gastric cancer (GC) [21]. However, the relations of PRGs with LGG are still unknown. Therefore, this work has explored the molecular characteristics and clinical significance of PRGs for patients with LGG.

## 2. Materials and Methods

### 2.1. Pathological Sampling

Paraffin-embedded tissue samples were gathered from 85 patients diagnosed with LGG at Zhongnan Hospital, Wuhan University, between May 2016 and March 2019. Clinicopathological data such as age, gender, grade, 1p/19q codeletion, IDH mutation status, and recurrence, together with the survival status of each patient, were collected until 31 December 2021. The inclusion criteria were as follows: (1) primary tumor tissue; (2) >18 years old; (3) pathological diagnosis of grade 2 or 3; (4) complete clinicopathological information; (5) corresponding tissue wax blocks available; and (6) patients followed-up until 31 December 2021. Patients conforming to the criteria below were excluded: (1) recurrent tumors; (2) <18 years of age; (3) incomplete clinicopathological information; (4) a lack of corresponding tissue blocks; (5) not followed-up to the specified deadline; and (6) patients with multiple simultaneous tumors. Our study protocols gained approval from the Ethics Committee of the Zhongnan Hospital of Wuhan University (ethics No. 2022169K). Additionally, the present study was performed following the Declaration of Helsinki.

### 2.2. Immunohistochemical Staining (IHC)

The expression of CASP4 and CASP9 was detected using IHC in 85 glioma tissue samples. First, we constructed tissue microarrays (TMAs). After dewaxing in xylene, rehydration in alcohol, and the blocking of endogenous peroxidase activity, TMAs were subjected to incubation overnight at 4 °C with specific antibodies against CASP4 (Abcam, AB25898, Waltham, MA, USA) or CASP9 (Abcam, AB202068, Waltham, MA, USA). The next day, a sheep anti-rabbit secondary antibody was supplemented, followed by incubation for half an hour at 25 °C. After DAB was added, color development was observed under a microscope. Finally, hematoxylin was added to counter-stain sections, followed by rinsing with water and dehydration with gradient alcohol as well as fixation using neutral gum sealing tablets. Positive cells in ×400 and ×100 microscopic fields were observed, and two pathologists unaware of the corresponding tissue information were responsible for assessing the IHC results.

### 2.3. IHC Evaluation

The gene expression levels within the tissues were assessed as follows: (1) This work chose five distinct fields of view (FOVs) at random under 400× magnification and rated positively stained cell scores as 0–4 for ≤5%, 6–25%, 26–50%, 51–75%, and >75%, respectively. (2) Staining intensity score: the immunohistochemistry staining intensities were rated as follows: negative (0 points), weak (1 point), intermediate (2 points), or high (3 points). Finally, the two scores were added: high and low expression of CASP4 and CASP9 were defined as scores of >6 and ≤6, respectively.

### 2.4. Data Sources

The present work acquired clinical information together with gene expression profiles (fragments per kilobase million, FPKM) of 523 LGG cases based on the TCGA database. Meanwhile, 1152 non-carcinoma brain tissues were obtained at the University of California Santa Cruz (UCSC) Xena website (https://xenabrowser.net/datapages/, accessed on 10 February 2022) using the iGTEx project. The GSE108474 dataset (Rembrandt, FPKM) was obtained from the GEO database (https://www.ncbi.nlm.nih.gov/geo/, accessed on 10 February 2022). Afterwards, clinical data along with RNA-seq data for additional LGG cases were also downloaded based on the Chinese Glioma Genome Atlas (CGGA) (http://www.cgga.org.cn/, accessed on 10 February 2022). Batch normalization was applied by using the ‘sva’ and ‘limma’ R packages. The downloaded FPKM data were converted to transcripts per kilobase million (TPM). Data from patients without prognostic and clinical information were excluded, resulting in a total of 811 LGG cases (TCGA: 518, Rembrandt: 121, and CGGA: 172) being included in the subsequent analyses. Details of the patients with LGG are presented in Table 1, including the clinical variables of age, gender, grade, 1p/19q codeletion, IDH mutation status, and overall survival time, together with the survival status.

### 2.5. Mutation and Copy Number Alteration (CNA) Analysis

The cBio Cancer Genomics Portal [22] (cBioPortal, http://www.cbioportal.org, accessed on 5 March 2022) integrates DNA methylation data, mRNA and microRNA profiles, non-synonymous mutations, protein expression levels, and DNA copy numbers from the TCGA, ICGC, GEO, and other databases. Using the cbioportal online website, we summarized the copy number and somatic mutation genetic variation of 33 PRGs in LGG. Mutations in the top 15 genes were mapped as an oncoplot.

### 2.6. Prognostic Risk Model Construction

For evaluating whether PRGs could be adopted for predicting the prognosis of patients with LGG, a univariate regression was used to examine the relationship of PRGs with TCGA-patient prognosis. We chose 19 survival-related genes with *p* < 0.05 in subsequent analyses. The R package “glmnet” function was adopted for constructing a prognosis prediction model by a least absolute shrinkage and selection operator (LASSO) Cox regression analysis [23]. TCGA-LGG cases were classified into high- or low-risk groups based on the median risk score value. Kaplan–Meier (KM) curves were generated and log-rank tests were carried out with the aim of analyzing the overall survival (OS) of both groups (“survival” and “survminer” R packages). Values of the area under the receiver operating characteristic (ROC) curve (AUC) were constructed based on the R package “pROC” function, with an aim of assessing the model prediction performance.

### 2.7. Tumor-Infiltrating Immune Cell (TIIC) Analysis

The TIIC levels *(n* = 24) in LGG tissues were examined using an ssGSEA approach [24] with the R software GSVA package (http://www.biocondutor.org/package/release/bioc/html/GSVA.html, accessed on 20 March 2022). In line with the signature genes reported for those 24 TIICs, we analyzed the TIIC enrichment levels of every gene in every tumor tissue based on the gene expression data. In addition, we conducted a Spearman’s correlation to identify the relations of the prognosis gene with every TIIC.

### 2.8. Single-Cell RNA-Seq

We obtained the single-cell RNA-seq dataset GSE182109 in the GEO database. Using <20% mitochondrial transcripts or 200 expressed genes as the threshold, we eliminated low-quality sequencing data. Seurat V4.0 [25], along with Harmony V1.0 [26], was adopted for the normalization and clustering as well as batch correction of single cells. Finally, 25,533 genes and 21,249 cells were retained and divided into different cell types to further evaluate the CASP4 expression in each type.

### 2.9. Construction of a Prognostic Nomogram

With the purpose of determining the 1-, 3-, and 5-year survival of individual patients, this work built a nomogram according to combined univariate and multivariate analysis results. A Cox proportional hazard (PH) test was conducted before constructing the nomogram (Supplementary Figure S1). To be specific, the nomogram that included clinical features markedly related to risk scores and calibration plots was constructed using the R package ‘RMS’. Nomogram-estimated probabilities were mapped against those measured values to assess the calibration curves, with the 45-degree line denoting the optimal prediction results. This work utilized the concordance index (C-index) for nomogram discrimination, and it was determined using 1000 bootstraps. In addition, the C-index was used to compare the nomogram prediction accuracy with single prognostic factors. Values of *p* < 0.05 (two-sided) were considered statistically significant.

### 2.10. GO/KEGG Pathway Analysis

To better understand PRG-related functions and pathways, this study utilized the R package “cluster Profiler” for a GO/KEGG analysis. Typically, GO terms can be categorized into biological process (BP) and molecular functions (MF), together with cellular component (CC) terms. KEGG has been developed as the database that integrates data on the genome, systemic functions, and chemistry. Adjusted values of *p* < 0.05 were considered statistically significant.

### 2.11. Gene Set Enrichment Analysis (GSEA)

The CASP4-expression-related pathways within LGG were predicted by a gene set enrichment analysis (GSEA) [27] (version 4.1) based on the TCGA-LGG dataset. According to the median CASP4 level, this work classified LGG samples into low- or high-expression groups. The Molecular Signatures Database (MSigDB, https://www.gsea-msigdb.org/gsea/msigdb, accessed on 10 February 2022) was adopted for downloading c2.cp.v7.2. symbols.gmt, the functional annotation dataset, as a control. GSEA enrichment was estimated using the normalized enrichment score (NES). In addition, the significance of the enrichment was evaluated at FDR < 0.25 and *p* < 0.05.

### 2.12. Statistical Analysis

R software v.3.6.3 was employed for the bioinformatic analysis. To be specific, the normal distribution of datasets was evaluated by Kolmogorov–Smirnov tests for the sake of determining if parametric or nonparametric rank-based analyses must be utilized. A Spearman correlation was utilized to assess correlation. For continuous and categorical data, the hypothesis was tested by the Wilcoxon rank-sum and Fisher’s exact tests, respectively. In addition, a survival analysis was conducted to examine the relation of patient features with their OS with a Cox proportional hazard model. “survival” in R was adopted for drawing Kaplan–Meier (KM) survival curves, while “survminer” was used to compare different subgroups with log-rank tests. “pROC” in R was utilized to obtain ROC curves, sensitivity, and specificity. Using SPSS22.0, this work evaluated the proportional hazard test within Cox models with Schoenfeld’s test, a KM analysis, and a log-log cumulative survival graph. *p* < 0.05 was thought to be statistically significant.

## 3. Results

### 3.1. PRG Expression in LGG

**Figure 1:**
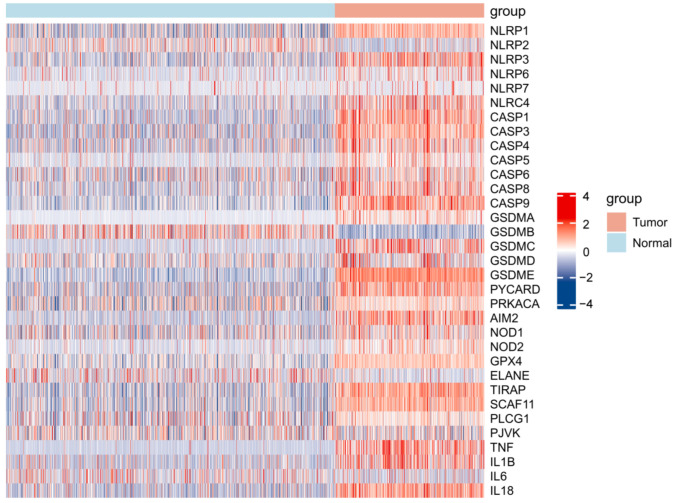
Heat map representing the levels of 33 PRGs within tumor and normal samples: red and blue stand for up- and down−regulation, respectively.

This work analyzed the levels of 33 PRGs within LGG and non-carcinoma brain samples. Gene levels in LGG were collected in the TCGA database, and normal tissue expression data were obtained from the GTEx database. The SVA package was adopted to remove batch effects. Altogether, 32 PRGs showed upregulation or downregulation within LGG, whereas there was no difference in the expression of PJVK (Figure 1).

**Figure 2:**
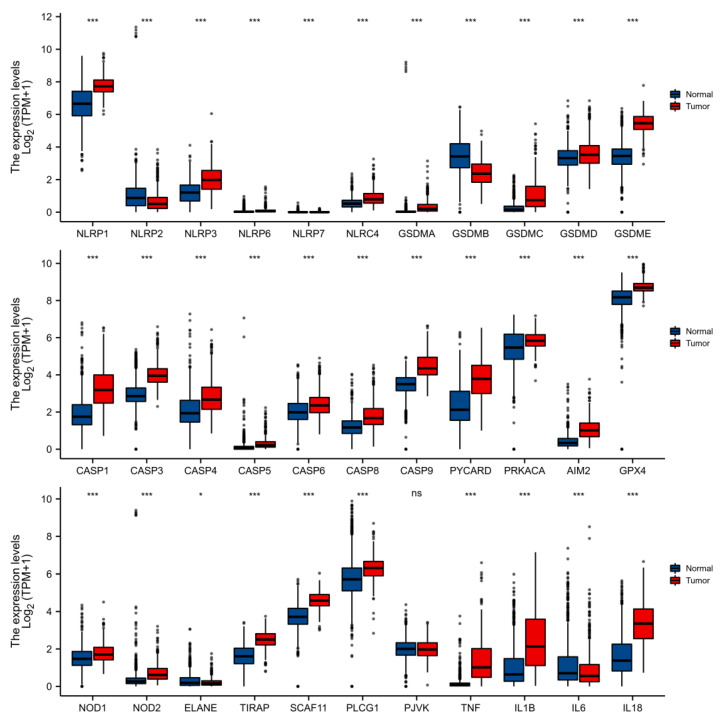
PRG levels within non-carcinoma (blue box) and cancer (red box) tissues in TCGA. * *p* ≤ 0.05; *** *p* ≤ 0.001; ns: have no statistical differences.

More specifically, the expression of NLRP1, NLRP3, NLRP6, NLRC4, CASP1, CASP3, CASP4, CASP5, CASP6, CASP8, CASP9, GSDMA, GSDMC, GSDMD, GSDME, PYCARD, PRKACA, AIM2, NOD1, NOD2, GPX4, TIRAP, SCAF11, PLCG1, TNF, IL1B, and IL18 was increased in LGG relative to non-carcinoma samples, whereas the expression of NLRP2, NLRP7, GSDMB, ELANE, and IL6 was decreased (Figure 2).

### 3.2. Genetic Variation and Functional Enrichment Analysis of PRGs in LGG

**Figure 3:**
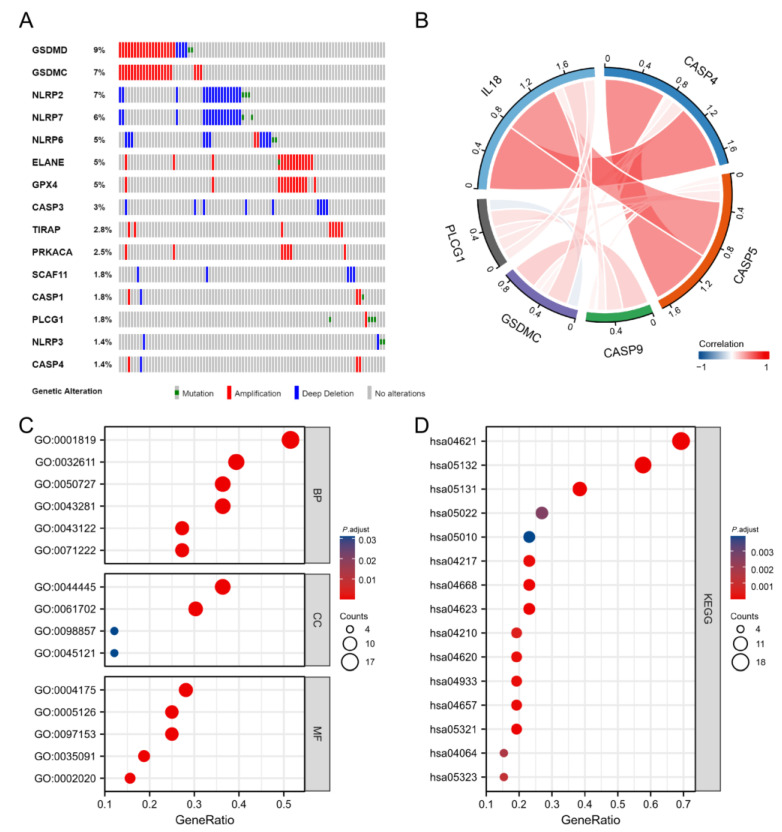
The genetic variation landscape and GO/KEGG analysis of PRGs in LGG. (**A**) Oncoplot of the top ten genes in PRGs. (**B**) Correlation of six genes incorporated into risk model. Red bars and blue bars are positive and negative associations, respectively, and the width indicates the degree of correlation. (**C**) BP, CC, and MF enrichment analysis results of PRGs. (**D**) The top 15 KEGG enrichment signaling pathways for PRGs.

Using the cBioPortal online tool (https://www.cbioportal.org/, accessed on 5 March 2022), the copy number and somatic mutation genetic variations were summarized for the 33 PRGs in LGG. Of the 283 samples, 101 (36%) showed genetic mutations. The five most significant genes in terms of mutation frequency included GSDMD, GSDMC, NLRP2, NLRP7, and NLRP6 (Figure 3A).

To clarify PRG functions, GO as well as KEGG databases were adopted to analyze their pathways. The 33 PRGs showed major relations to BP terms such as interleukin-1 beta production, the regulation of I-κB kinase/NF-κB signaling, and the regulation of cysteine-type endopeptidase activity involved in the apoptotic process; MF terms, including cytokine receptor binding and cysteine-type endopeptidase activity involved in the apoptotic process; and CC terms such as the cytosolic part and inflammasome complex (Figure 3C). According to KEGG enrichment, the 33 PRGs showed major involvement in the NF-κB pathway, NOD-like receptor pathway, pathways of neurodegeneration, and the toll-like receptor pathway as well as the TNF pathway (Figure 3D).

### 3.3. PRG Prognostic Model Establishment

For constructing the prognostic gene model, this study first used a univariate regression to identify prognostic PRGs, then performed a LASSO Cox regression to construct the prognostic gene model on the basis of these prognostic PRGs. Risk score= $CASP4\times \left(0.70905\right)+CASP5\times \left(-0.89475\right)$ $+CASP9\times \left(-0.39372\right)+GSDMC\times \left(-0.21076\right)$ $+PLCG1\times \left(0.75315\right)+IL18\times \left(0.28316\right)$. The relationships amongst these six genes are shown in Figure 3B.

**Figure 4:**
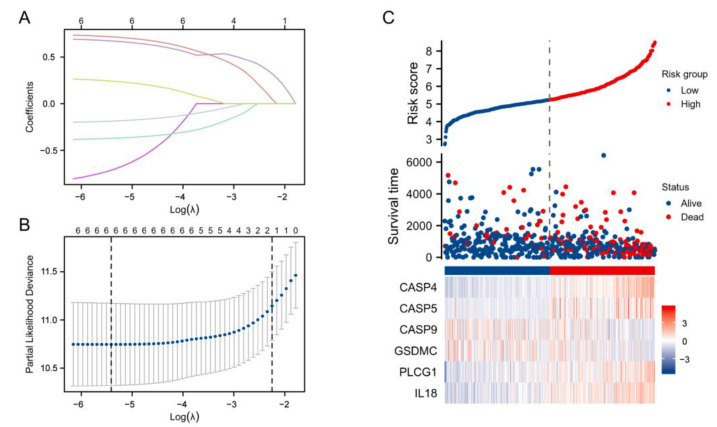
Construction of a prognostic model for pyroptosis-related genes: (**A**) LASSO variable trajectory plot. (**B**) LASSO coefficient filter. (**C**) Distribution of risk scores, survival status, and the expression of six prognostic indicators in LGG patients.

**Figure 5:**
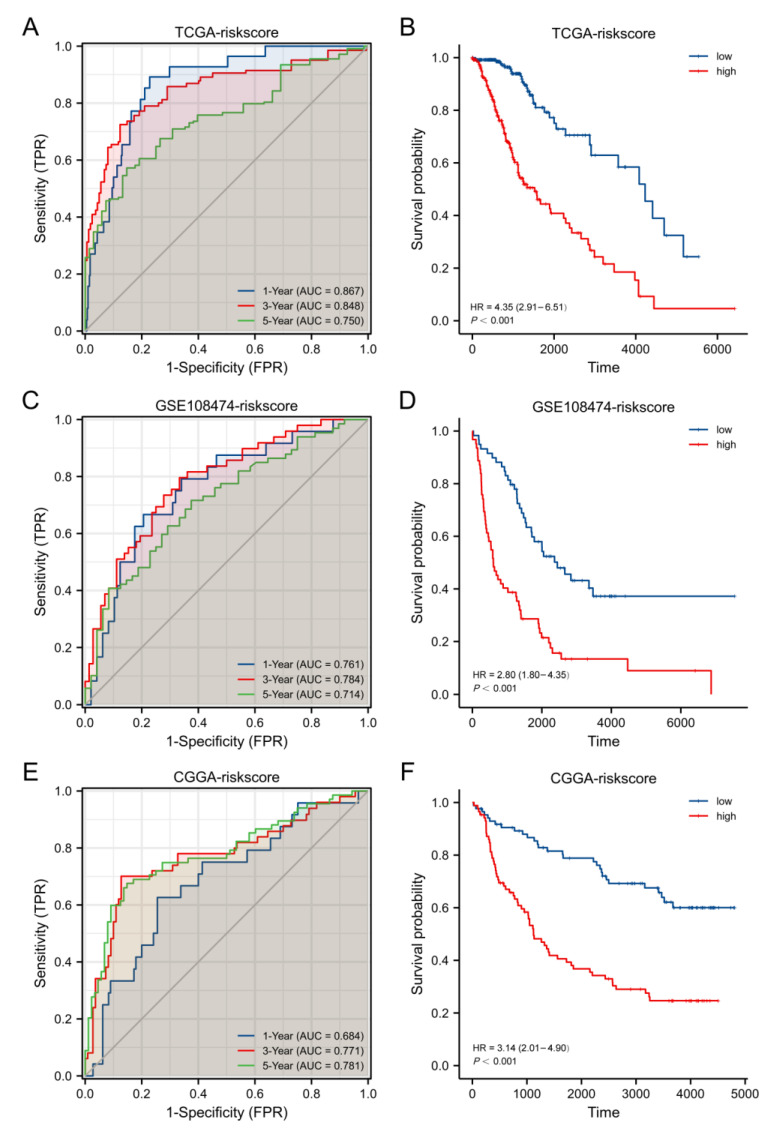
Prognosis prediction performance of our constructed risk score model for LGG patients: Kaplan–Meier plots and ROC curves for the TCGA (**A**,**B**), GSE108474 (**C**,**D**), and CGGA datasets (**E**,**F**).

According to the risk score, we divided the LGG cases into two subgroups. With the increase in risk score, patient mortality risk also elevated accordingly, while OS time was shortened (Figure 4C). Based on the KM analysis, LGG cases in the high-risk-score group exhibited a relationship to the poor OS when compared with the low-risk-score group (median OS time, 4.2 years vs. 11.6 years, *p* < 0.001, Figure 5A). The model sensitivity and specificity were later assessed with a time-dependent ROC analysis, with 1-, 3-, and 5-year survival AUC values of 0.867, 0.848, and 0.750, respectively (Figure 5B).

### 3.4. Validation with External Datasets

This work chose RNA sequence datasets from LGG patients in the CGGA (CGGA325, Grade II: 98 and Grade III: 74) and GSE108474 (Grade II: 64 and Grade III: 57) datasets for prognosis prediction model validation. The risk scores of all cases were determined. Later, cases were divided into low- or high-risk subgroups according to the median score and compared using the ROC and KM curves of different groups. In the GSE108474 and CGGA datasets, the KM analysis suggested that survival rates notably declined in high-risk subgroups relative to low-risk subgroups (all *p* < 0.001, Figure 5D,F). As revealed by the ROC curve analysis, the constructed model exhibited high prognosis prediction performance in the GSE108474 (AUC = 0.761, 0.784, and 0.714 for 1-, 3-, and 5-year survivals) and CGGA (AUC = 0.684, 0.771, and 0.781 for 1-, 3-, and 5-year survivals) datasets (Figure 5C,E).

### 3.5. Independent Prognostic Values for the Prognostic Model

**Figure 6:**
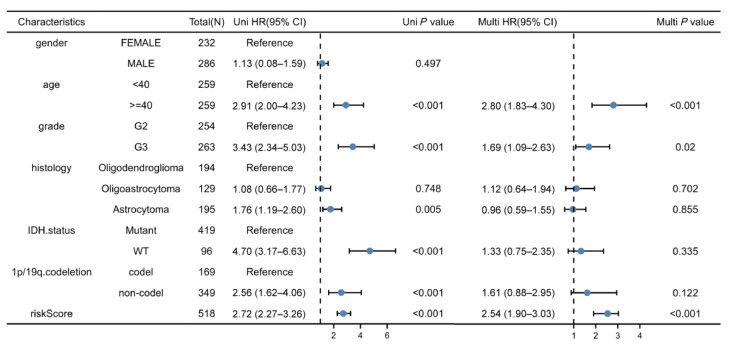
Univariate as well as multivariate regressions of clinicopathological factors and risk scores in TCGA–LGG cases.

Univariate as well as multivariate regressions were conducted for evaluating if our gene-model-derived risk scores could independently predict LGG prognostic outcomes. According to the univariate regression results, the ages, grades, histological types, 1p/19q codeletions, IDH mutation status, and risk scores of patients impacted their survival times. As revealed by the multivariate regression, the ages, grades, and risk scores of patients (hazard ratio = 2.54, *p* < 0.001) independently predicted LGG patient prognostic outcomes (all *p* < 0.05, Figure 6).

### 3.6. Relations of Clinicopathological Factors with the Prognostic Model

**Figure 7:**
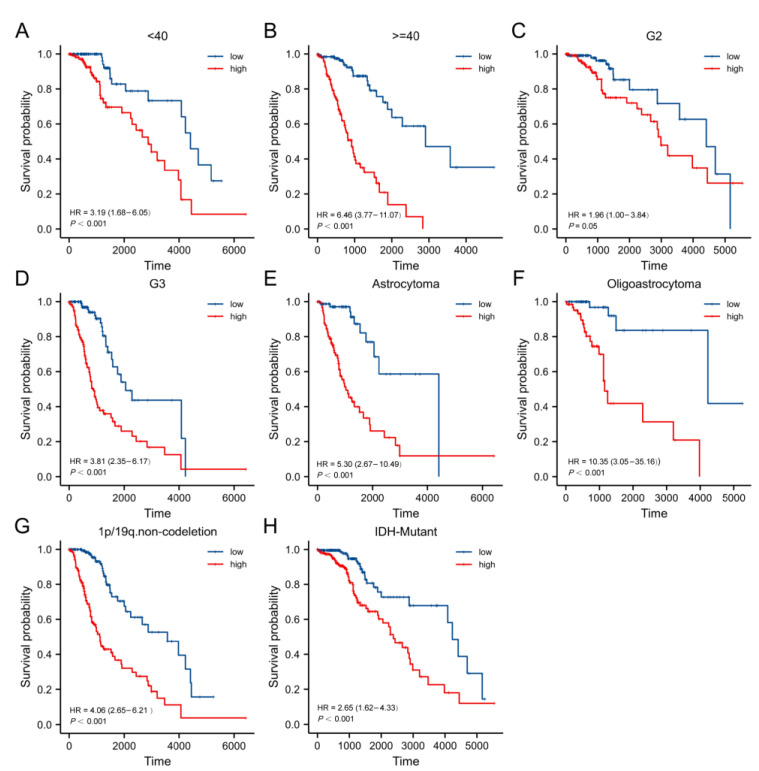
Kaplan–Meier curves of high- and low-risk cases with diverse clinicopathological subgroups: (**A**,**B**) age, (**C**,**D**) grade, (**E**,**F**) histology, (**G**) 1p19q non-codeletion, and (**H**) IDH mutation status.

This work evaluated our PRGs model’s accuracy in the prognosis of patients with diverse clinicopathological factors. Based on the KM analysis, high-risk cases had a worse prognosis in the <40 years (*p* < 0.001), ≥40 years (*p* < 0.001), grade 2 (*p* = 0.05), grade 3 (*p* < 0.001), astrocytoma (*p* < 0.001), oligoastrocytoma (*p* < 0.001), 1p/19q non-codeletion (*p* < 0.001), and IDH mutant (*p* < 0.001) subgroups (Figure 7).

### 3.7. Construction of a Predictive Nomogram

**Figure 8:**
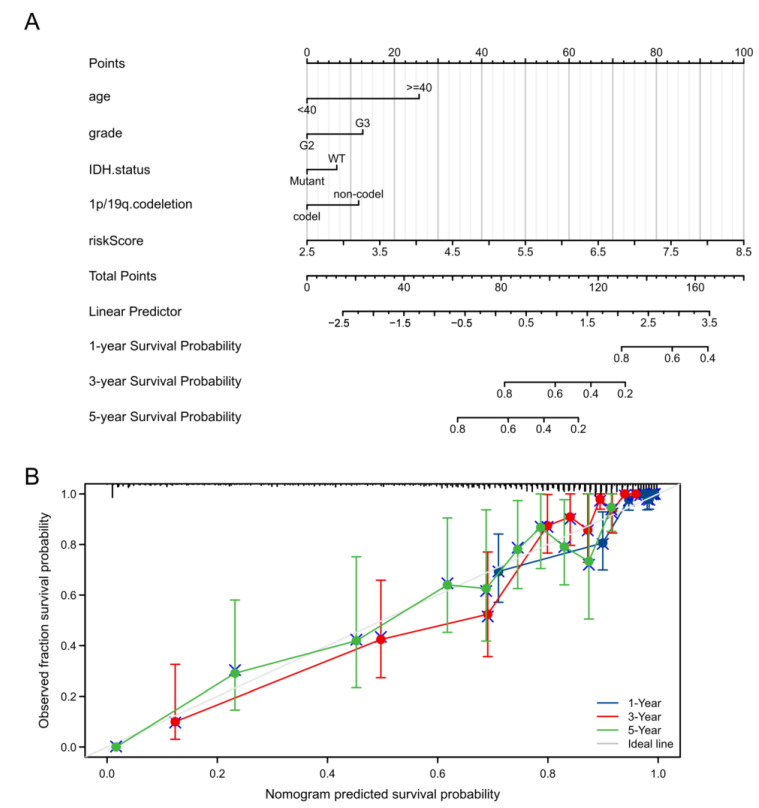
Predictive nomogram construction. (**A**) Proposed nomogram to predict 1-, 3-, and 5-year OS for LGG. (**B**) Calibration curves to evaluate the fit of the actual probabilities to the probabilities predicted by the model (C-index = 0.837). The diagonal line represents the ideal nomogram.

This study built a nomogram for the prediction of 1-, 3-, and 5-year OS rates in LGG by incorporating clinicopathological factors and the prognosis model. Our predictive nomogram performed well in the prediction of 3- and 5-year OS relative to the best model for the whole cohort, with a C-index of 0.837 (Figure 8).

### 3.8. PRGs Related to TIICs within LGG

Pyroptosis has an important effect on tumor immune microenvironment (TIME) development. According to our analysis, the PRGs related to prognosis (CASP4, CASP5, CASP9, GSDMC, PLCG1, and IL18) were correlated with TIICs within LGG.

**Figure 9:**
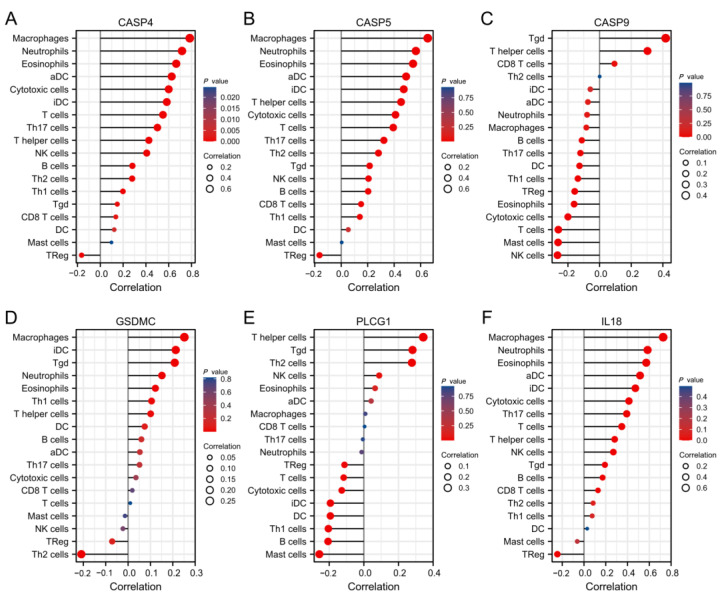
Correlation of model genes with multiple immune cell infiltration fractions (**A**–**F**). The sizes and lengths of the circles indicate the degree of correlation, and the orientation indicates whether the correlation is positive or negative.

As a result, CASP4, CASP5, and IL18 expression showed positive relations to the abundances of macrophages, neutrophils, and eosinophils (*p* < 0.001), and a negative relation to regulatory T-cell abundance (*p* < 0.001). CASP9 expression was positively related to the T gamma delta infiltration level and negatively related to the NK cell infiltration level (*p* < 0.001). The PLCG1 level was positively related to the T-helper cell level (Figure 9, *p* < 0.001).

### 3.9. CASP4 and CASP9 Are Independent Prognostic Factors for LGG

**Figure 10:**
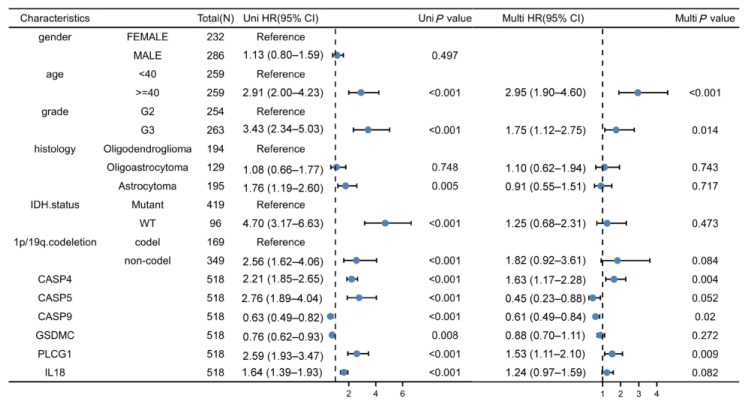
Univariate and multivariate regression of clinicopathological factors and model-related genes among TCGA-LGG cases.

This work utilized a Cox proportional hazards regression model of clinicopathological factors and risk genes. As a result, age, grade, CASP4, CASP9, and PLCG1 could all be independent prognostic factors for patients with LGG (Figure 10). Since PLCG1 has previously been shown as a prognostic marker for LGG cases [28], the present work only investigated the effects of CASP4 and CASP9.

### 3.10. High CASP4 Level Predicted Dismal Prognostic Outcomes

**Figure 11:**
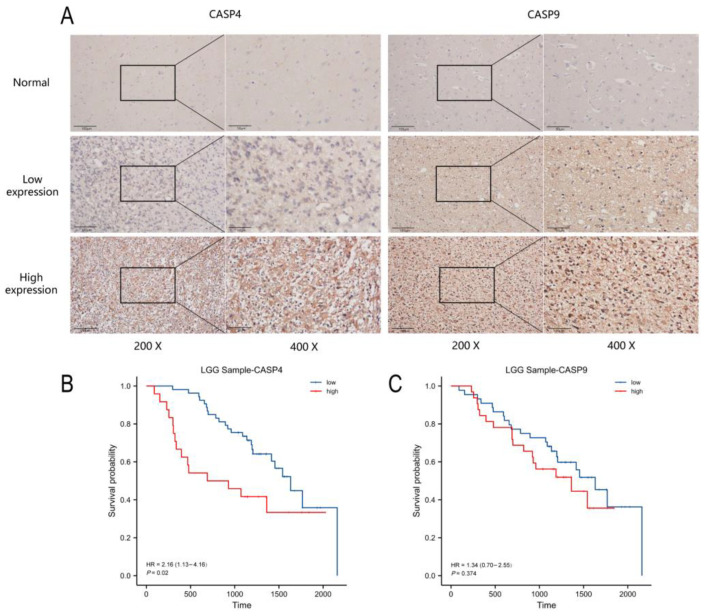
The group with high CASP4 expression showed poor prognostic outcomes: (**A**) IHC results in normal and LGG tissues. (**B**,**C**) Kaplan–Meier curves of CASP4 and CASP9 of 85 LGG cases.

As revealed by immunohistochemistry, the CASP4 and CASP9 levels in LGG were remarkably increased when compared with healthy brain samples (Figure 11A). Based on their expression, LGG cases were classified into low- or high-expression groups. On the basis of KM curves, CASP4 served as a prognostic indicator in patients with LGG (Figure 11B). There was no obvious difference in the survival time distribution of the low- and high-CASP9-expression groups (Figure 11C).

Univariate as well as multivariate regressions were then conducted using the clinicopathological information for patients from our hospital, and the results showed that grade (*p* = 0.01), CASP4 (*p* = 0.005), and IDH mutation (*p* < 0.001) independently predicted LGG patient prognosis (Table 2).

### 3.11. The Predictive Power of CASP4 Performed Well in Multiple LGG Databases

**Figure 12:**
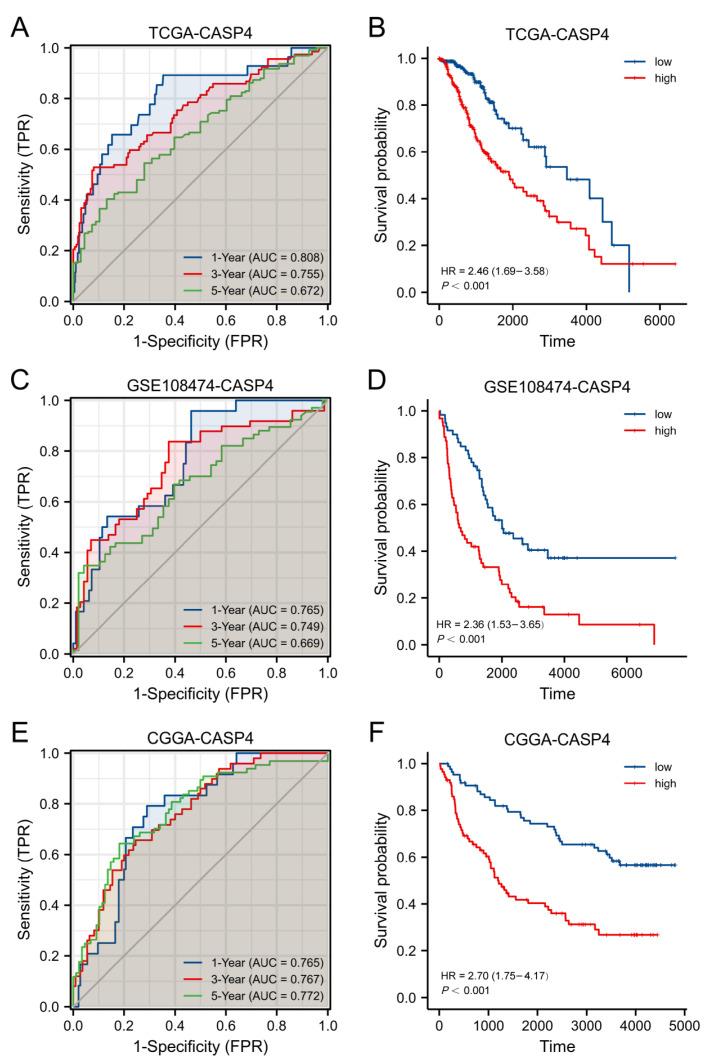
High CASP4 expression indicates dismal prognostic outcomes in LGG cases. LGG patient prognosis by Kaplan–Meier plots and ROC curves: (**A**,**B**) TCGA, (**C**,**D**) GSE108474, (**E**,**F**) CGGA.

The TCGA, GSE108474, and CGGA databases were adopted to verify the prognostic ability of CASP4. Based on the median CASP4 expression, LGG cases were classified into low- or high-expression groups. As revealed by the KM analysis, CASP4 with high expression had a poor prognosis compared with the low-expression group in all three databases (Figure 12B,D,F, *p* < 0.001). According to the ROC curves, CASP4 was effective in predicting prognosis in the TCGA (AUC = 0.808, 0.755, and 0.672 for 1-, 3-, 5-year survival), GSE108474 (AUC = 0.765, 0.749, and 0.669 for 1-, 3-, 5-year survival), and CGGA (AUC = 0.765, 0.767, and 0.772 for 1-, 3-, 5-year survival) databases (Figure 12A,C,E).

### 3.12. TIIC Levels Increased in High-CASP4-Expression Patients

**Figure 13:**
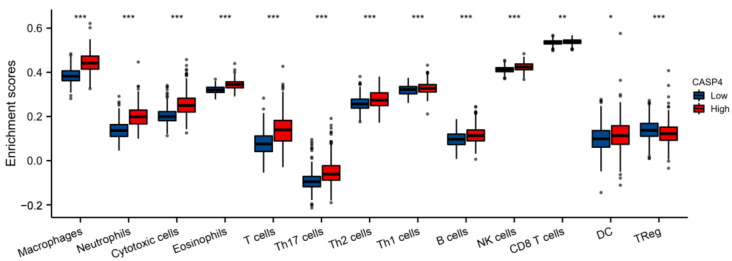
Immune infiltration scores in the high-CASP4-expression (red box) and low-CASP4-expression groups (blue box). * *p* ≤ 0.05; ** *p* ≤ 0.01; *** *p* ≤ 0.001.

To further analyze the relation of TIIC levels with CASP4 expression in the prognosis prediction model, this work conducted an ssGSEA with the R package ‘GSVA’ to quantify the functions and pathway levels of immune cells among LGG patients. Our findings demonstrated that the high-CASP4 -expression group had more macrophages, NK cells, and T-cell infiltration (Figure 13).

### 3.13. Single-Cell Analysis of CASP4

**Figure 14:**
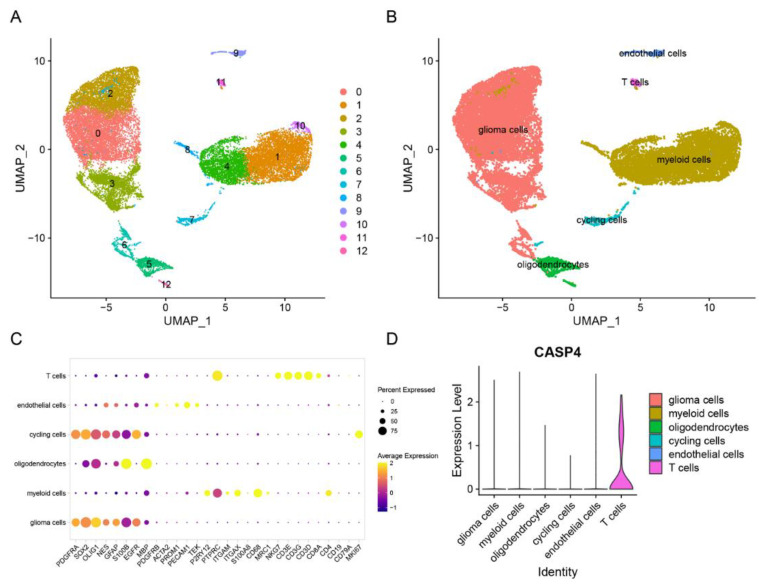
Single-cell analysis of CASP4: (**A**) A 2D UMP plot suggested the division of 21,249 single cells into 13 clusters, which are presented with diverse colors. (**B**) These 13 clusters were defined as six cell types. (**C**) Bubble diagram of marker genes for six cell types. (**D**) Expression of CASP4 in various cell types.

The ‘Seurat’ R package was utilized to process raw data. Later, cells were divided into 13 clusters (Figure 14A), which ultimately defined them as six cell types: oligodendrocytes, glioma, myeloid cells, astrocytes, T cells, cycling cells, and endothelial cells (Figure 14B). A violin plot was constructed, and it showed that CASP4 was predominantly expressed in T cells, indicating that CASP4′s role was possibly associated with alterations in the immune microenvironment (Figure 14D).

### 3.14. Functional Enrichment Analysis of CASP4

**Figure 15:**
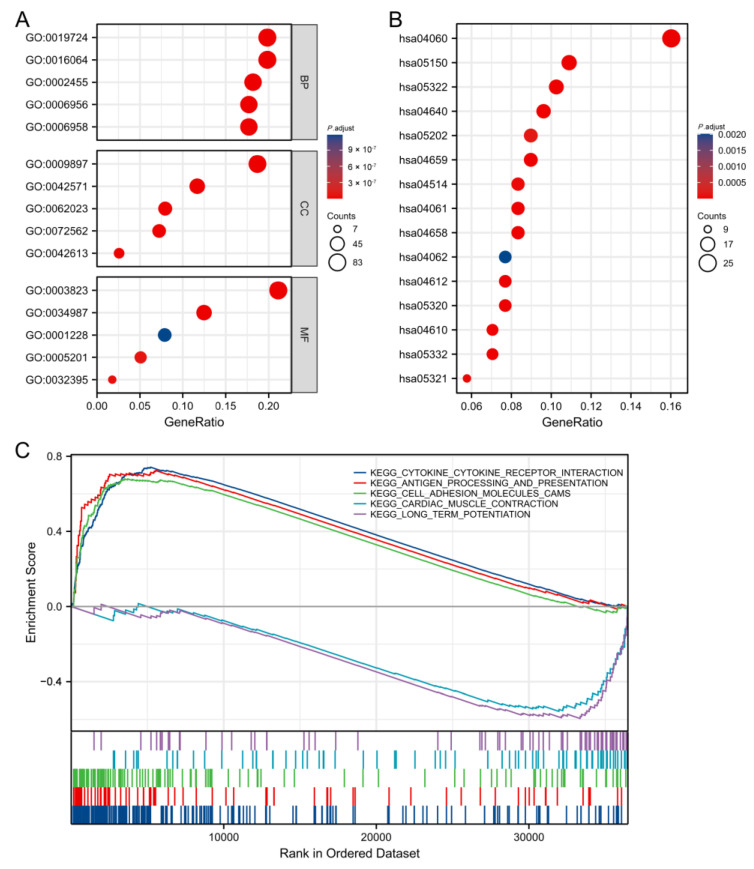
GO/KEGG analysis of the high- and low-CASP4-expression groups: (**A**) BP, CC, and MF enrichment analysis results. (**B**) The top 15 KEGG enrichment signaling pathways for CASP4. (**C**) GSEA enrichment analysis: select pathways with adjusted *p* < 0.05.

GO/KEGG analyses were performed to explore the differences in biological function and the related signal transduction pathways in the high- and low-CASP4-expression groups. The DEGs were mainly involved in the following BP terms: the immunoglobulin-mediated immune response, B-cell-mediated immunity, classical pathways, and complement activation; the following MF terms: extracellular matrix structural constituents and antigen binding; and the following CC terms: immunoglobulin circulation and the external side of the plasma membrane (Figure 15A). The GSEA showed that, in the high-CASP4-expression group, cytokine receptor interaction, cell adhesion molecule cams, antigen processing, and presentation gene sets were all significantly enriched. The long-term potentiation and cardiac muscle contraction gene sets were associated with the low-CASP4-expression group (Figure 15C).

## 4. Discussion

Currently, surgical resection, radiotherapy, and chemotherapy are the available clinical therapeutic options for LGG. However, these treatments do not significantly increase the patient survival rate [29]. As one of the histological subtypes of glioma, LGG still lacks effective prognostic markers [30].

Pyroptosis is a novel PCD triggered by proinflammatory signaling [14]. Some studies indicate that pyroptosis suppresses tumor occurrence and progression, but additional studies suggest that this proinflammatory type of cell death enhances TME development and thus cancer cell proliferation; however, its mechanisms are not fully understood and require further elucidation [10,31].

In this work, 33 PRGs were collected through a literature review. Their expression and prognostic roles in patients with LGG were elucidated. This work constructed a novel prognostic model using a LASSO analysis, and it included six PRGs, namely CASP4, CASP5, CASP9, GSDMC, PLCG1, and IL18. Recent studies have shown that genes related to cuproptosis [32,33] and ferroptosis [34,35] are predictive of the prognosis of LGG patients, and corresponding prognostic models have been constructed. Compared with them, our model has good 1-, 3-, 5- year AUC values (Supplementary Table S1).

CASP4 has previously been implicated in tumor genesis and progression, which has a variety of biological roles. For example, CASP4 upregulation is related to better OS in esophageal squamous cell carcinoma (ESCC) and GC [36,37], whereas in RCCC and NSCLC upregulation is associated with poor prognoses [20,38]. There are significantly different CASP4 levels in diverse tumors, as shown in the above studies. Further research is required to determine whether it is a tumor-promoting or tumor-suppressing factor. CASP5 acts on GSDMD, causing cell membrane pore generation [39]. After activation, CASP5 interacts with CASP1 to promote the activation, which cleaves the IL-1/IL-18 precursors to obtain active IL-1β/IL-18. In addition, these cytokines can be produced via channels generated through GSDMD-cNTs, inducing pyroptosis [40]. CASP5 is reportedly related to different cancers, such as cervical cancer, osteosarcoma, lung cancer, and human glioblastoma [41,42,43]. CASP9 may be involved in multiple cancers, autoimmune disorders, and neurological diseases [44]. miR-23a, miR-24a, and miR-582-5p reportedly function in colorectal cancer and glioblastoma by regulating CASP9 [45,46]. Polymorphisms in several introns and promoter regions of CASP9 are associated with the incidence and progression of lung, breast, liver, esophageal, leukemia, and other cancers [47,48]. At first, GSDMC shows high expression within metastatic melanoma cells, so it is consequently called melanoma-derived leucine zipper-containing extranuclear factor (MLZE) [49]. GSDMC gene knockout decreases colorectal cancer (CRC) cell growth, supporting the tumor-promoting effects of GSDMC [50]. However, GSDMC was found in one work to be suppressed within multiple ESCC cell lines, indicating its role as a tumor suppressor [51]. PLCG1 is an important second messenger in KDR signaling in endothelial cells [52]. Studies have shown that PLCG1 is related to the invasive ability of various malignant tumors, such as liver, lung, and prostate cancer, but its relationship with tumor cell proliferation remains controversial [28,53,54]. IL-18 belongs to the IL-1 family, and like IL-1β, it can be processed via caspase 1 as the active cytokine. It is related to macrophages, Th1/Th2, NK, and IL-17-producing γδ T cell activation, and it rapidly produces many cytokines that can kill tumor cells [55,56]. A recent in vitro study demonstrated that immune checkpoint therapy with IL-18 combined with anti-PD-L1 or anti-CTLA4 inhibited tumor progression [57].

Recent research has revealed functional crossovers between pyroptosis, apoptosis, and necroptosis [58]. GSDME can be cleaved and activated by key molecules involved in apoptosis, thereby regulating changes in the membrane permeability of late apoptotic cells, which is a form of pyroptosis [59]. Membrane disruption by MLKL (an end-effector for necroptosis) activates NLRP3, thus initiating pyroptosis. The link between different PCDs will require further investigation [60].

The KM and ROC curve analyses of our prognostic model showed good prediction results in the TCGA, GSE108474, and CGGA databases. As revealed by a multivariate regression, the risk score can independently predict LGG patient prognosis. Subsequently, to further evaluate the LGG patient survival time, this study combined our prognostic model with clinicopathological factors to construct a nomogram, and the results showed that our nomogram model performed well in predicting OS rates for LGG cases at 1, 3, and 5 years.

Upon univariate/multivariate regression with clinicopathological factors and model genes, CASP4, CASP9, and PLCG1 could act as independent prognostic factors for LGG. Since PLCG1 was already known to be associated with LGG patient prognostic outcomes [28], we further studied CASP4 and CASP9. Using clinical tissue samples and follow-up information, we confirmed using IHC that high-CAGSP4-expression cases exhibited poorer prognostic outcomes.

Cancer immunotherapy based on immune checkpoint inhibitors (ICIs) has shown significant clinical success in recent years [61], despite the fact that only one third of patients with most forms of cancer respond to these medications [62]. Recent research has demonstrated that pyroptosis can synergistically increase the antitumor effectiveness of ICIs, which may be related to pyroptosis altering the immunological microenvironments of tumors [63,64]. Pyroptosis-related genes were connected to the extracellular matrix, a crucial component of the tumor microenvironment, according to the GO/KEGG results. Its components and their interactions with glioma cells affect tumor invasiveness [65]. We carried out an enrichment analysis on PRGs and found that they were mostly involved in the NOD-like receptor, tumor necrosis factor, toll-like receptor, and NF-κB signaling pathways. These pathways are tightly related to glioma genesis and development [66,67,68]. Pyroptosis-induced inflammation in the tumor environment can activate immune cells and immune factors, thereby enhancing the efficiency of cancer immunotherapy [9]. In vitro studies have shown that PD-1/PD-L1 inhibitors can restore the activity of antitumor T cells, make tumor cells regress, and improve patient survival [69,70]. According to a single-cell analysis, CASP4 showed high expression within T cells. B cells, T cells, NK cells, and macrophages as well as other TIIC levels in high-CASP4-expression patients increased, indicating that it can modulate tumor genesis and development by changing the TME. We can thus see that pyroptosis-related genes have great potential in immunotherapy.

However, this study has certain limitations. First, we only adopted online databases for analysis, and the prognostic model was not verified by additional experiments. Second, the number of clinical specimens was not large, and limited by the experimental conditions, we could only verify the prognostic effects of CASP4 by IHC; consequently, further investigations will be required to understand its mechanism of action.

## 5. Conclusions

The present work establishes a PRG-based prognostic model that can predict LGG patient prognosis and offers a certain reference for clinical decision making. A study on the PRGs included in our prognostic model was also conducted. As a result, LGG cases with high CASP4 expression showed reduced survival time. This indicates that CASP4 can independently predict LGG patient prognosis. Its effect on immune cell infiltration may thus offer more guidance for immunotherapy.

## Figures and Tables

**Table 1:** Baseline characteristics of LGG patients in three databases.

Characteristic	TCGA	GSE108474	CGGA325
Total, N	518	121	172
Gender, n (%)			
Female	232 (29.5%)	37 (4.7%)	66 (8.4%)
Male	286 (36.3%)	60 (7.6%)	106 (13.5%)
Age, n (%)			
<40	247 (30.6%)	50 (6.2%)	88 (10.9%)
≥40	271 (33.6%)	67 (8.3%)	84 (10.4%)
Grade, n (%)			
G2	254 (31.4%)	64 (7.9%)	98 (12.1%)
G3	263 (32.5%)	57 (7%)	74 (9.1%)
IDH status, n (%)			
Mutant	419 (61.1%)	NA	127 (18.5%)
Wildtype	96 (14%)	NA	44 (6.4%)
1p/19q Codeletion, n (%)			
codeletion	169 (23.8%)	8 (1.1%)	55 (7.8%)
non-codeletion	349 (49.2%)	13 (1.8%)	115 (16.2%)

**Table 2:** Univariate and multivariate regressions of the clinicopathological information of 85 LGG patients.

Characteristics	Total (N)	Univariate Regression		Multivariate Regression	
		Hazard Ratio (95% CI)	*p*-Value	Hazard Ratio (95% CI)	*p*-Value
Age	85				
<40	29	Reference			
≥40	56	1.725 (0.87–3.42)	0.118		
Grade	85				
G2	47	Reference			
G3	38	2.175 (1.20–3.96)	0.011	2.566 (1.25–5.27)	0.010
IDH status	76		<0.001		
Mutant	53	Reference			
WT	23	4.970 (2.56–9.65)	<0.001	5.889 (2.45–14.17)	<0.001
TERT mutation	77		0.412		
Mutant	27	Reference			
WT	50	1.336 (0.67–2.67)	0.412		
MGMT methylation	77		0.018		
high	53	Reference			
low	24	2.217 (1.15–4.29)	0.018	1.219 (0.57–2.59)	0.606
1p19q Codeletion	77		0.006		
codeletion	24	Reference			
non-codeletion	53	3.709 (1.44–9.53)	0.006	1.656 (0.49–5.56)	0.414
CASP4 expression	77		0.020		
low	53	Reference			
high	24	2.165 (1.13–4.16)	0.020	3.062 (1.40–6.70)	0.005

## Data Availability

Publicly available datasets were analyzed in this study. These data can be found on the following sites: the RNA-seq data consisting of LGG samples from the University of California Santa Cruz (UCSC) Xena website (https://xenabrowser.net/datapages/, accessed on 10 February 2022), the Chinese Glioma Genome Atlas (CGGA) (http://www.cgga.org.cn/, accessed on 10 February 2022), and the GEO database (https://www.ncbi.nlm.nih.gov/geo/, accessed on 10 February 2022).

## Supplementary Materials

The following supporting information can be downloaded at: https://www.mdpi.com/article/10.3390/cells11243980/s1, Figure S1: Cox proportional hazards (PH) test before constructing the nomogram; Table S1: Comparison of AUC values of different prognostic models based on TCGA-LGG data. References [32,33,34,35] are cited in Supplementary Materials

## References

[1] Louis D.N., Perry A., Reifenberger G., von Deimling A., Figarella-Branger D., Cavenee W.K., Ohgaki H., Wiestler O.D., Kleihues P., Ellison D.W. (2016). The 2016 World Health Organization Classification of Tumors of the Central Nervous System: A Summary. Acta Neuropathol..

[2] Gittleman H., Sloan A.E., Barnholtz-Sloan J.S. (2020). An Independently Validated Survival Nomogram for Lower-Grade Glioma. Neuro-Oncol..

[3] Ostrom Q.T., Patil N., Cioffi G., Waite K., Kruchko C., Barnholtz-Sloan J.S. (2020). Cbtrus Statistical Report: Primary Brain and Other Central Nervous System Tumors Diagnosed in the United States in 2013–2017. Neuro-Oncol..

[4] Lombardi G., Barresi V., Castellano A., Tabouret E., Pasqualetti F., Salvalaggio A., Cerretti G., Caccese M., Padovan M., Zagonel V. (2020). Clinical Management of Diffuse Low-Grade Gliomas. Cancers.

[5] Jones J., Nguyen H., Drummond K., Morokoff A. (2021). Circulating Biomarkers for Glioma: A Review. Neurosurgery.

[6] Ludwig K., Kornblum H.I. (2017). Molecular Markers in Glioma. J. Neurooncol..

[7] Bedoui S., Herold M.J., Strasser A. (2020). Emerging Connectivity of Programmed Cell Death Pathways and Its Physiological Implications. Nat. Rev. Mol. Cell Biol..

[8] Moujalled D., Strasser A., Liddell J.R. (2021). Molecular Mechanisms of Cell Death in Neurological Diseases. Cell Death Differ..

[9] Tan Y., Chen Q., Li X., Zeng Z., Xiong W., Li G., Li X., Yang J., Xiang B., Yi M. (2021). Pyroptosis: A New Paradigm of Cell Death for Fighting against Cancer. J. Exp. Clin. Cancer Res..

[10] Du T., Gao J., Li P., Wang Y., Qi Q., Liu X., Li J., Wang C., Du L. (2021). Pyroptosis, Metabolism, and Tumor Immune Microenvironment. Clin. Transl. Med..

[11] Loveless R., Bloomquist R., Teng Y. (2021). Pyroptosis at the Forefront of Anticancer Immunity. J. Exp. Clin. Cancer Res..

[12] Tsuchiya K. (2021). Switching from Apoptosis to Pyroptosis: Gasdermin-Elicited Inflammation and Antitumor Immunity. Int. J. Mol. Sci..

[13] Zhou Z., He H., Wang K., Shi X., Wang Y., Su Y., Wang Y., Li D., Liu W., Zhang Y. (2020). Granzyme a from Cytotoxic Lymphocytes Cleaves Gsdmb to Trigger Pyroptosis in Target Cells. Science.

[14] Thi H.T.H., Hong S. (2017). Inflammasome as a Therapeutic Target for Cancer Prevention and Treatment. J. Cancer Prev..

[15] Fang Y., Tian S., Pan Y., Li W., Wang Q., Tang Y., Yu T., Wu X., Shi Y., Ma P. (2020). Pyroptosis: A New Frontier in Cancer. Biomed. Pharmacother..

[16] Lin W., Chen Y., Wu B., Chen Y., Li Z. (2021). Identification of the Pyroptosisrelated Prognostic Gene Signature and the Associated Regulation Axis in Lung Adenocarcinoma. Cell Death Discov..

[17] Shen Y., Li X., Wang D., Zhang L., Li X., Xia T., Shang X., Yang X., Su L., Fan X. (2021). Novel Prognostic Model Established for Patients with Head and Neck Squamous Cell Carcinoma Based on Pyroptosis-Related Genes. Transl. Oncol..

[18] Li X.Y., Zhang L.Y., Li X.Y., Yang X.T., Su L.X. (2021). A Pyroptosis-Related Gene Signature for Predicting Survival in Glioblastoma. Front. Oncol..

[19] Ye Y., Dai Q., Qi H. (2021). A Novel Defined Pyroptosis-Related Gene Signature for Predicting the Prognosis of Ovarian Cancer. Cell Death Discov..

[20] Meng L., Tian Z., Long X., Diao T., Hu M., Wang M., Zhang W., Zhang Y., Wang J., He Y. (2020). Caspase 4 Overexpression as a Prognostic Marker in Clear Cell Renal Cell Carcinoma: A Study Based on the Cancer Genome Atlas Data Mining. Front. Genet..

[21] Zhou C.B., Fang J.Y. (2019). The Role of Pyroptosis in Gastrointestinal Cancer and Immune Responses to Intestinal Microbial Infection. Biochim. Biophys. Acta Rev. Cancer.

[22] Gao J., Aksoy B.A., Dogrusoz U., Dresdner G., Gross B., Sumer S.O., Sun Y., Jacobsen A., Sinha R., Larsson E. (2013). Integrative Analysis of Complex Cancer Genomics and Clinical Profiles Using the Cbioportal. Sci. Signal.

[23] Friedman J., Hastie T., Tibshirani R. (2010). Regularization Paths for Generalized Linear Models Via Coordinate Descent. J. Stat. Softw..

[24] Bindea G., Mlecnik B., Tosolini M., Kirilovsky A., Waldner M., Obenauf A.C., Angell H., Fredriksen T., Lafontaine L., Berger A. (2013). Spatiotemporal Dynamics of Intratumoral Immune Cells Reveal the Immune Landscape in Human Cancer. Immunity.

[25] Satija R., Farrell J.A., Gennert D., Schier A.F., Regev A. (2015). Spatial Reconstruction of Single-Cell Gene Expression Data. Nat. Biotechnol..

[26] Korsunsky I., Millard N., Fan J., Slowikowski K., Zhang F., Wei K., Baglaenko Y., Brenner M., Loh P.R., Raychaudhuri S. (2019). Fast, Sensitive and Accurate Integration of Single-Cell Data with Harmony. Nat. Methods.

[27] Hänzelmann S., Castelo R., Guinney J. (2013). Gsva: Gene Set Variation Analysis for Microarray and Rna-Seq Data. BMC Bioinform..

[28] Li T., Yang Z., Li H., Zhu J., Wang Y., Tang Q., Shi Z. (2022). Phospholipase Cgamma1 (Plcg1) Overexpression Is Associated with Tumor Growth and Poor Survival in Idh Wild-Type Lower-Grade Gliomas in Adult Patients. Lab. Investig..

[29] Brat D.J., Verhaak R.G., Aldape K.D., Yung W.K., Salama S.R., Cooper L.A., Rheinbay E., Miller C.R., Vitucci M., Cancer Genome Atlas Research Network (2015). Comprehensive, Integrative Genomic Analysis of Diffuse Lower-Grade Gliomas. N. Engl. J. Med..

[30] Pienkowski T., Kowalczyk T., Kretowski A., Ciborowski M. (2021). A Review of Gliomas-Related Proteins. Characteristics of Potential Biomarkers. Am. J. Cancer Res..

[31] Xia X., Wang X., Cheng Z., Qin W., Lei L., Jiang J., Hu J. (2019). The Role of Pyroptosis in Cancer: Pro-Cancer or Pro-Host?. Cell Death Dis..

[32] Miao Y., Liu J., Liu X., Yuan Q., Li H., Zhang Y., Zhan Y., Feng X. (2022). Machine Learning Identification of Cuproptosis and Necroptosis-Associated Molecular Subtypes to Aid in Prognosis Assessment and Immunotherapy Response Prediction in Low-Grade Glioma. Front. Genet..

[33] Yan X., Wang N., Dong J., Wang F., Zhang J., Hu X., Zhao H., Gao X., Liu Z., Li Y. (2022). A Cuproptosis-Related Lncrnas Signature for Prognosis, Chemotherapy, and Immune Checkpoint Blockade Therapy of Low-Grade Glioma. Front. Mol. Biosci..

[34] Zhao J., Liu Z., Zheng X., Gao H., Li L. (2021). Prognostic Model and Nomogram Construction Based on a Novel Ferroptosis-Related Gene Signature in Lower-Grade Glioma. Front. Genet..

[35] Huang Q.R., Li J.W., Yan P., Jiang Q., Guo F.Z., Zhao Y.N., Mo L.G. (2022). Establishment and Validation of a Ferroptosis-Related Lncrna Signature for Prognosis Prediction in Lower-Grade Glioma. Front. Neurol..

[36] Wang Z., Ni F., Yu F., Cui Z., Zhu X., Chen J. (2019). Prognostic Significance of Mrna Expression of Casps in Gastric Cancer. Oncol. Lett..

[37] Shibamoto M., Hirata H., Eguchi H., Sawada G., Sakai N., Kajiyama Y., Mimori K. (2017). The Loss of Casp4 Expression Is Associated with Poor Prognosis in Esophageal Squamous Cell Carcinoma. Oncol. Lett..

[38] Terlizzi M., Colarusso C., de Rosa I., de Rosa N., Somma P., Curcio C., Sanduzzi A., Micheli P., Molino A., Saccomanno A. (2018). Circulating and Tumor-Associated Caspase-4: A Novel Diagnostic and Prognostic Biomarker for Non-Small Cell Lung Cancer. Oncotarget.

[39] Broz P., Pelegrin P., Shao F. (2020). The Gasdermins, a Protein Family Executing Cell Death and Inflammation. Nat. Rev. Immunol..

[40] Kayagaki N., Stowe I.B., Lee B.L., O’Rourke K., Anderson K., Warming S., Cuellar T., Haley B., Roose-Girma M., Phung Q.T. (2015). Caspase-11 Cleaves Gasdermin D for Non-Canonical Inflammasome Signalling. Nature.

[41] Zhang Y., He R., Lei X., Mao L., Jiang P., Ni C., Yin Z., Zhong X., Chen C., Zheng Q. (2021). A Novel Pyroptosis-Related Signature for Predicting Prognosis and Indicating Immune Microenvironment Features in Osteosarcoma. Front. Genet..

[42] Zhou Y., Dai W., Wang H., Pan H., Wang Q. (2018). Long Non-Coding Rna Casp5 Promotes the Malignant Phenotypes of Human Glioblastoma Multiforme. Biochem. Biophys. Res. Commun..

[43] Babas E., Ekonomopoulou M.T., Karapidaki I., Doxakis A., Betsas G., Iakovidou-Kritsi Z. (2010). Indication of Participation of Caspase-2 and Caspase-5 in Mechanisms of Human Cervical Malignancy. Int. J. Gynecol. Cancer.

[44] Avrutsky M.I., Troy C.M. (2021). Caspase-9: A Multimodal Therapeutic Target with Diverse Cellular Expression in Human Disease. Front. Pharmacol..

[45] Floyd D.H., Zhang Y., Dey B.K., Kefas B., Breit H., Marks K., Dutta A., Herold-Mende C., Synowitz M., Glass R. (2014). Novel Anti-Apoptotic Micrornas 582-5p and 363 Promote Human Glioblastoma Stem Cell Survival Via Direct Inhibition of Caspase 3, Caspase 9, and Bim. PLoS ONE.

[46] Shang J., Yang F., Wang Y., Wang Y., Xue G., Mei Q., Wang F., Sun S. (2014). Microrna-23a Antisense Enhances 5-Fluorouracil Chemosensitivity through Apaf-1/Caspase-9 Apoptotic Pathway in Colorectal Cancer Cells. J. Cell. Biochem..

[47] Jeong H.-S., Hye Y.C., Lee E.-R., Kim J.-H., Jeon K., Lee H.-L., Cho S.-G. (2011). Involvement of Caspase-9 in Autophagy-Mediated Cell Survival Pathway. Biochim. Et Biophys. Acta BBA Mol. Cell Res..

[48] Kim B., Srivastava S.K., Kim S.-H. (2015). Caspase-9 as a Therapeutic Target for Treating Cancer. Expert Opin. Ther. Targets.

[49] Watabe K., Ito A., Asada H., Endo Y., Kobayashi T., Nakamoto K., Itami S., Takao S., Shinomura Y., Aikou T. (2001). Structure, Expression and Chromosome Mapping of Mlze, a Novel Gene Which Is Preferentially Expressed in Metastatic Melanoma Cells. Jpn. J. Cancer Res..

[50] Miguchi M., Hinoi T., Shimomura M., Adachi T., Saito Y., Niitsu H., Kochi M., Sada H., Sotomaru Y., Ikenoue T. (2016). Gasdermin C Is Upregulated by Inactivation of Transforming Growth Factor Beta Receptor Type Ii in the Presence of Mutated Apc, Promoting Colorectal Cancer Proliferation. PLoS ONE.

[51] Saeki N., Usui T., Aoyagi K., Kim D.H., Sato M., Mabuchi T., Yanagihara K., Ogawa K., Sakamoto H., Yoshida T. (2009). Distinctive Expression and Function of Four Gsdm Family Genes (Gsdma-D) in Normal and Malignant Upper Gastrointestinal Epithelium. Genes Chromosom. Cancer.

[52] Jang H.J., Suh P.G., Lee Y.J., Shin K.J., Cocco L., Chae Y.C. (2018). Plcgamma1: Potential Arbitrator of Cancer Progression. Adv. Biol. Regul..

[53] Shin K.J., Jang H.J., Lee Y.J., Lee Y.G., Suh P.G., Yang Y.R., Chae Y.C. (2021). Phospholipase Cgamma1 Represses Colorectal Cancer Growth by Inhibiting the Wnt/Beta-Catenin Signaling Axis. Biochem. Biophys. Res. Commun..

[54] Tang W., Zhou Y., Sun D., Dong L., Xia J., Yang B. (2019). Oncogenic Role of Phospholipase C-Gamma1 in Progression of Hepatocellular Carcinoma. Hepatol. Res..

[55] Kaplanski G. (2018). Interleukin-18: Biological Properties and Role in Disease Pathogenesis. Immunol. Rev..

[56] Yasuda K., Nakanishi K., Tsutsui H. (2019). Interleukin-18 in Health and Disease. Int. J. Mol. Sci..

[57] Ma Z., Li W., Yoshiya S., Xu Y., Hata M., El-Darawish Y., Markova T., Yamanishi K., Yamanishi H., Tahara H. (2016). Augmentation of Immune Checkpoint Cancer Immunotherapy with Il18. Clin. Cancer Res..

[58] Snyder A.G., Oberst A. (2021). The Antisocial Network: Cross Talk between Cell Death Programs in Host Defense. Annu. Rev. Immunol..

[59] Wang Y., Kanneganti T.D. (2021). From Pyroptosis, Apoptosis and Necroptosis to Panoptosis: A Mechanistic Compendium of Programmed Cell Death Pathways. Comput. Struct. Biotechnol. J..

[60] Gutierrez K.D., Davis M.A., Daniels B.P., Olsen T.M., Ralli-Jain P., Tait S.W., Gale M., Oberst A. (2017). Mlkl Activation Triggers Nlrp3-Mediated Processing and Release of Il-1β Independently of Gasdermin-D. J. Immunol..

[61] Wang Y., Zhang H., Liu C., Wang Z., Wu W., Zhang N., Zhang L., Hu J., Luo P., Zhang J. (2022). Immune Checkpoint Modulators in Cancer Immunotherapy: Recent Advances and Emerging Concepts. J. Hematol. Oncol..

[62] Jiang P., Gu S., Pan D., Fu J., Sahu A., Hu X., Li Z., Traugh N., Bu X., Li B. (2018). Signatures of T Cell Dysfunction and Exclusion Predict Cancer Immunotherapy Response. Nat. Med..

[63] Reck M., Schenker M., Lee K.H., Provencio M., Nishio M., Lesniewski-Kmak K., Sangha R., Ahmed S., Raimbourg J., Feeney K. (2019). Nivolumab Plus Ipilimumab Versus Chemotherapy as First-Line Treatment in Advanced Non-Small-Cell Lung Cancer with High Tumour Mutational Burden: Patient-Reported Outcomes Results from the Randomised, Open-Label, Phase Iii Checkmate 227 Trial. Eur. J. Cancer.

[64] Tang R., Xu J., Zhang B., Liu J., Liang C., Hua J., Meng Q., Yu X., Shi S. (2020). Ferroptosis, Necroptosis, and Pyroptosis in Anticancer Immunity. J. Hematol. Oncol..

[65] Ferrer V.P., Neto V.M., Mentlein R. (2018). Glioma Infiltration and Extracellular Matrix: Key Players and Modulators. Glia.

[66] Saxena S., Jha S. (2017). Role of Nod- Like Receptors in Glioma Angiogenesis: Insights into Future Therapeutic Interventions. Cytokine Growth Factor Rev..

[67] Xue L., Lu B., Gao B., Shi Y., Xu J., Yang R., Xu B., Ding P. (2019). Nlrp3 Promotes Glioma Cell Proliferation and Invasion Via the Interleukin-1β/Nf-Κb P65 Signals. Oncol. Res..

[68] Xu J., Zhang Z., Qian M., Wang S., Qiu W., Chen Z., Sun Z., Xiong Y., Wang C., Sun X. (2020). Cullin-7 (Cul7) Is Overexpressed in Glioma Cells and Promotes Tumorigenesis Via Nf-Κb Activation. J. Exp. Clin. Cancer Res..

[69] Ladomersky E., Zhai L., Lenzen A., Lauing K.L., Qian J., Scholtens D.M., Gritsina G., Sun X., Liu Y., Yu F. (2018). Ido1 Inhibition Synergizes with Radiation and Pd-1 Blockade to Durably Increase Survival against Advanced Glioblastoma. Clin. Cancer Res..

[70] Kim J.E., Patel M.A., Mangraviti A., Kim E.S., Theodros D., Velarde E., Liu A., Sankey E.W., Tam A., Xu H. (2017). Combination Therapy with Anti-Pd-1, Anti-Tim-3, and Focal Radiation Results in Regression of Murine Gliomas. Clin. Cancer Res..

