# Supplementary material for: Systematic Analysis of a Pyroptosis-Related Signature to Predict the Prognosis and Immune Microenvironment of Lower-Grade Glioma
Source: Cells. 2022 Dec 9;11(24):3980. doi: 10.3390/cells11243980 (PMC9776729; doi:10.3390/cells11243980)
Supplement: Supplementary file 1 [file cells-11-03980-s001.zip › cells-2049004-supplementary.pdf]

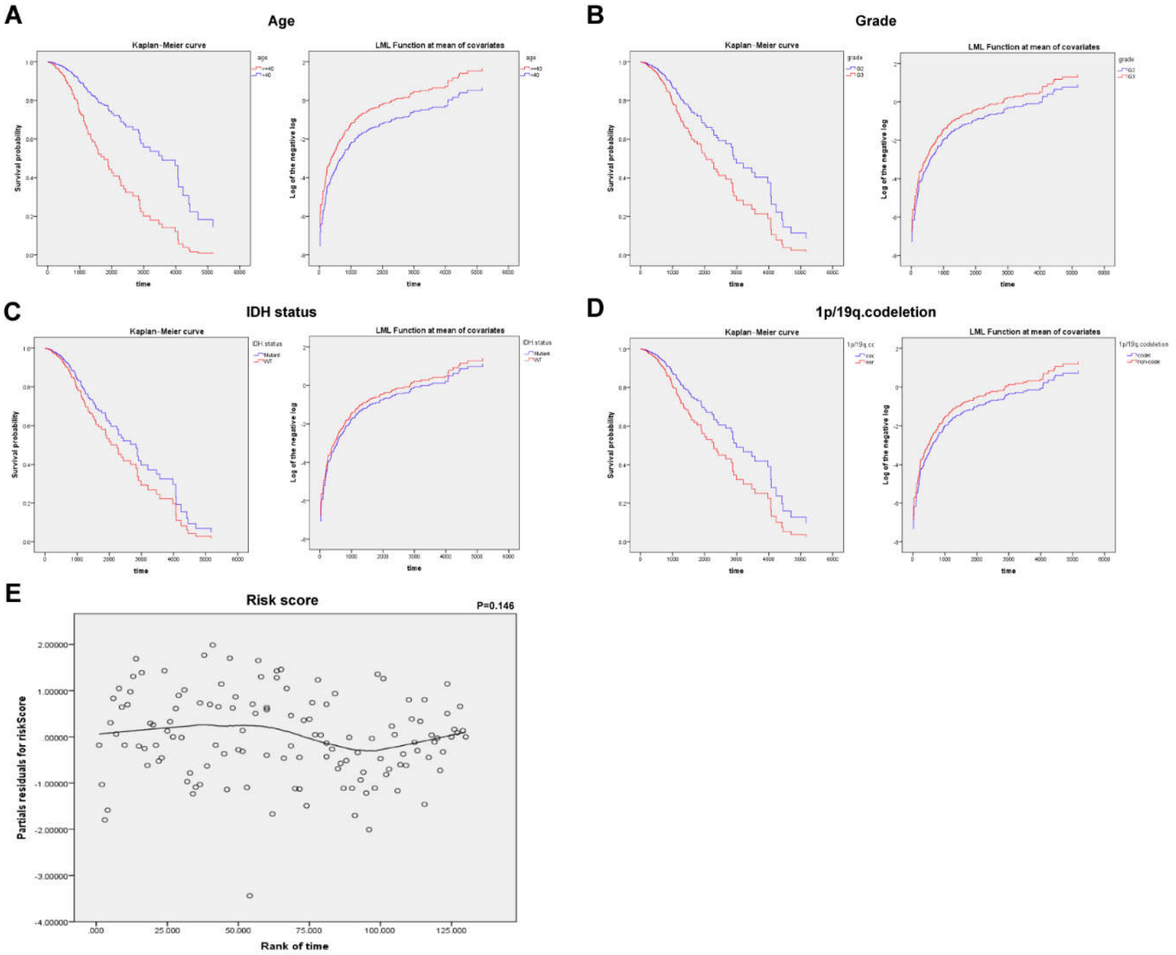

Supplementary Figure S1. Cox proportional hazard (PH) test was conducted before constructing the nomogram. (A-D) Schoenfeld's test showed that age, grade, IDH mutation status, and 1p/19q codeletion met the PH test. (E) Schoenfeld's test showed that the pyroptosis-related risk score met PH test ($p > 0.05$).

Table S1. Comparison of AUC values of different prognostic models based on TCGA-LGG data

**Publication**	**Types of prognostic models**	**AUC values**		
		**1-year**	**3-year**	**5-year**
Ours	Pyroptosis	0.867	0.848	0.750
Miao et al., 2022 [32]	Cuproptosis	0.787	0.824	0.760
Yan et al., 2022 [33]	Cuproptosis, LncRNA	0.841	0.839	0.768
Zhao et al., 2021 [34]	Ferroptosis	0.855	0.834	0.753
Huang et al., 2022 [35]	Ferroptosis, LncRNA	0.893	0.811	0.719
